# Clinical outcomes of sub-millisievert CT evaluation of congenital chest wall deformity using photon counting CT: a new standard of care?

**DOI:** 10.21203/rs.3.rs-9334962/v1

**Published:** 2026-04-27

**Authors:** Joseph Cao, Ananya Gupta, Steve Bache, Justin Solomon, Kayla Kilpatrick, Tamara N. Fitzgerald, Charles Maxfield, Michael Fadell, Caroline Carrico, Ana Gaca, Kristina Hallam, Maragatha Kuchibhatla, Megan Maloney, Ryan Antiel, Sara Janos

**Affiliations:** Duke University School of Medicine; Duke University School of Medicine; Duke University School of Medicine; Duke University School of Medicine; Duke University School of Medicine; Duke University School of Medicine; Duke University School of Medicine; Duke University School of Medicine; Duke University School of Medicine; Duke University School of Medicine; Siemens Healthineers (United States); Duke University School of Medicine; Duke University School of Medicine; Duke University School of Medicine; Duke University School of Medicine

**Keywords:** Child, Thoracic Wall, Radiation Exposure, Clinical Protocols, Tomography, X-Ray Computed, Radiation Dosage, Radiology

## Abstract

**Background:**

Pectus excavatum is the most common congenital chest wall deformity and is typically treated surgically. Disease severity is commonly assessed using the CT-derived Haller Index (HI), creating opportunities to reduce radiation exposure in pediatric patients. Photon-counting detector CT (PCD-CT) enables substantial dose reduction; however, the clinical impact of sub-millisievert (sub-mSv) imaging has not been well established.

**Objective:**

To evaluate the impact of sub-millisievert (sub-mSv) PCD-CT scans on clinical outcomes in the preoperative assessment of congenital pectus deformities.

**Methods:**

This retrospective study included patients with congenital pectus deformities treated at a single institution. Introduction of a PCD-CT system and dose-optimized protocols enabled comparison between standard-dose (SD) and sub-mSv CT cohorts. Demographic and clinical characteristics, including age, body mass index (BMI), HI, and effective diameter, were compared, along with radiation exposure metrics and postoperative clinical outcomes.

**Results:**

There were no significant differences between the SD and sub-mSv cohort in age (unadjusted/adjusted p = 0.32/1), HI (unadjusted p = 0.27/1), BMI (unadjusted p = 0.44/1), or effective diameter (unadjusted p = 0.22/1). Median sub-mSv protocol CTDI_vol_, DLP, SSDE, and effective dose were 0.03 mGy, 1.35 mGy*cm, 0.04 mGy, and 0.06 mSv, respectively. Radiation dose was significantly lower than the SD group (Hodges-Lehmann estimate (95% C.I.): effective dose − 2.10 (−2.88, −1.28), SSDE − 6.66 (−8.55, −4.08); unadjusted and adjusted p < 0.001 for both). Postoperative complication rates were low and did not significantly differ between cohorts in the immediate post-operative period or at outpatient follow up (unadjusted and adjusted p = 1 for both).

**Conclusion:**

Sub-mSv PCD-CT provides diagnostic image quality for preoperative evaluation of congenital chest wall deformities while reducing radiation exposure to levels comparable to a chest radiograph. These findings support broader adoption of low-dose PCD-CT in pediatric chest imaging and emphasize the importance of outcome-based validation of dose reduction strategies.

## Introduction

Pectus excavatum is the most common congenital chest wall abnormality [1]. It typically presents in early childhood, resulting from unbalanced growth of the costochondral cartilage, which leads to either symmetric or asymmetric deformities of the anterior chest wall. Disease severity ranges from asymptomatic cosmetic symptoms to dyspnea, chest pain, and clinically significant cardiovascular disease [2–4]. Treatment is usually surgical with open or minimally invasive techniques using a convex steel bar [5, 6]. The Haller Index (HI), originally defined on chest computed tomography (CT) as the ratio of maximum transverse diameter of the chest to minimum anterior-posterior diameter from the sternum to the spine, remains the most widely used method for assessing the degree of chest wall deformity [7]. The primary concern with the diagnostic work-up in pediatric patients is the radiation dose associated with conventional CT imaging.

Magnetic resonance (MR) imaging has proven effective for evaluating chest wall deformities without ionizing radiation and offers the added benefit of cardiac function assessment [8–10]. Despite these advantages, MRI remains limited by chest wall motion artifacts, which can significantly alter measurements. Recent studies have shown that respiratory motion can affect HI and correction index (CI) calculations across clinically significant thresholds affecting treatment decisions [11]. Differentials in MRI access along with payer constraints further limit its broader adoption.

Efforts to reduce radiation exposure in pectus assessment have also validated the use of radiography and reduced field-of-view CT [12, 13]. Nevertheless, CT remains the standard of care for pre-surgical evaluation because of its ability to characterize sternal position relative to the anterior rib cage and its ability to calculate the CI to assess potential degree of operative repair [14, 15]. Radiation dose reduction, particularly in pediatric CT imaging has been a cornerstone of pediatric radiology [16]. Studies on reducing chest CT radiation dose have been conducted for various indications in adult and pediatric patients studying parenchymal disease and bony abnormalities [17–20]. The commercial release of photon-counting detector CT (PCD-CT) in 2022 introduced a new generation of low-dose CT imaging, achieving the greatest dose reductions in chest imaging [21–23].

To our knowledge, no studies have characterized postoperative outcomes using either of these chest CTs. As such, this study aims to describe and compare postoperative outcomes in patients undergoing sub-millisievert (sub-mSv) chest CT versus standard-dose chest CT for preoperative assessment of pectus excavatum. We hypothesize that a significantly reduced radiation dose non-contrast chest CT does not adversely affect operative outcomes in the evaluation and surgical management of congenital chest wall deformities.

## Materials and Methods

Study protocols were approved by the Institutional Review Board at [*institution withheld*]; informed consent for retrospective chart review was waived. A reduced radiation dose protocol was implemented in our department for evaluation of the bony thorax following installation of a first-generation photon counting CT (PCD-CT) (Siemens NAEOTOM Alpha; Siemens Healthineers, Forchheim, Germany). Protocols were designed based on prior work that investigated dose reduction in pediatric chest imaging [21, 24]. All imaging was performed at a tertiary care children’s hospital.

### Patient Selection

**Figure 1:**
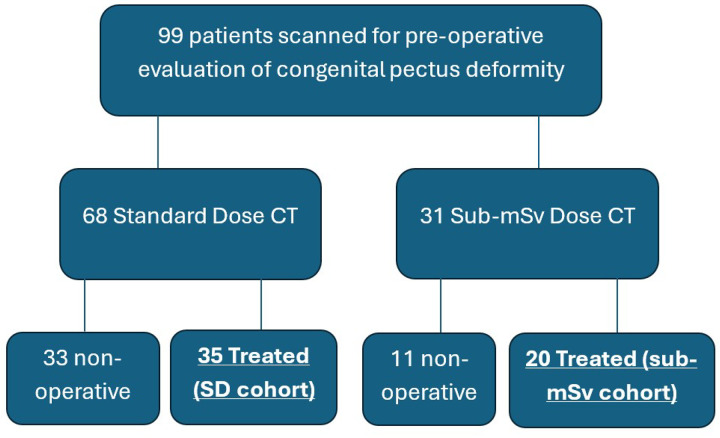
Patient identification and cohort assignment.

Study participants were selected retrospectively based on clinical diagnoses of chest wall deformity and clinical referral for pre-operative evaluation using non-contrast CT of the chest. Inclusion criteria consisted of patients up to 20 years of age who obtained a non-contrast chest CT for pre-surgical planning, underwent surgical treatment, and completed post-operative outpatient follow-up. Patients who met the inclusion criteria were identified retrospectively through the electronic medical record from 2017 –2025 (Figure 1). The standard dose (SD) control cohort is represented by patients who underwent a standard dose non-contrast CT of the chest as their pre-operative scan. Patients scanned using the reduced dose protocol, which was developed on the PCD-CT, comprised the sub-mSv cohort. Patient factors that may be associated with clinical outcomes were examined, including patient age and body mass index (BMI), as well as the HI are included in Table 1 [25]. Patient effective diameter was calculated from the CT scout image.

### CT Acquisition, Reconstruction Parameters, and Dose Tracking

Standard radiation dose non-contrast chest CTs were performed on 2^nd^ and 3^rd^ generation EID-CTs (SOMATOM Flash/Force, Siemens Healthineers, Forchheim, Germany). Sub-mSv scans were performed on a first-generation PCD-CT (NAEOTOM Alpha, Siemens Healthineers, Forchheim, Germany) protocols and reconstruction parameters for which are outlined in Table 2. CT scan adequacy was determined by the reading radiologist tasked with assessing degree of chest wall deformity which included measuring the HI and CI. [15]. Radiation dose metrics including CT dose index (CTDI_vol_) and dose length product (DLP) were obtained from the dose report. Size specific dose estimate (SSDE) was calculated based on AAPM task group report 204 methodology. Effective dose calculations were performed using anatomy and age-based conversion factors [26].

### Patient Outcomes

Patient outcomes were reviewed in the immediate post-operative period and at the outpatient post-operative follow-up visit. A composite of known potential complications was scored based on previously reported post-operative complications including seroma, hematoma, pneumonia, chronic pain, persistent effusion, persistent pneumothorax, bar dislodgement, and bar infection [27]. No complications, non-operative complications, and complications requiring repeat surgical intervention were scored as 0, 1, and 2 respectively. Length of post-operative stay was recorded for both cohorts.

### Statistical Analysis

Study population characteristics including age, HI, BMI, and effective diameter were compared between SD and sub-mSv cohorts using Wilcoxon rank sum tests (age, HI, effective diameter) or a two-sample t-test with equal variances (BMI), depending on normality. Immediate post-operative and outpatient follow-up clinical outcomes were compared using Fisher’s exact tests due to small cell counts. Length of stay was compared between both cohorts using a Wilcoxon rank sum test due to non-normally distributed data. Effective dose and SSDE between both cohorts were also compared using a Wilcoxon rank sum test due to non-normally distributed data.

Differences between the cohorts (sub-mSv minus SD) are reported with Hodges-Lehmann estimates for non-normally distributed variables (age, HI, effective diameter, length of stay, effective dose, and SSDE) or the difference in means for normally distributed variables (BMI), along with 95% confidence intervals (C.I.). All variables are presented with the raw, unadjusted p-values, and adjusted p-values using the Holm method. All analyses were conducted using SAS 9.4 (SAS Institute Inc., Cary, NC).

## Results

### Patient Population

Between 2017 to 2025, 99 patients received non-contrast CTs of the chest for pre-surgical evaluation of pectus excavatum. Fifty-five patients underwent surgical correction for congenital pectus deformity and completed outpatient post-operative follow-up (inclusion group). Thirty-five operative patients (63.6%) received the standard dose non-contrast CT (SD cohort). Twenty operative patients (36.4%) received the sub-mSv scan (sub-mSv cohort). Cohort selection is outlined in Figure 1. Of the excluded patients (11/31) scanned in the sub-mSv cohort, 4 did not complete pre-surgical evaluation, 3 surgeries were cancelled due to insurance denial, and 3 patients were not surgical candidates based on age or disease severity. One surgery was aborted prior to bar placement unrelated to imaging findings.

Most individuals in both groups were male, with 33 (94.3%) in the SD group and 16 (80%) in the sub-mSv group. The mean age was similar between both groups, with means of 15.96 (Std Dev: 1.98) years in the SD cohort and 15.34 (Std Dev: 1.75) years in the sub-mSv cohort. The mean BMI of the SD group was 19.22 (Std Dev: 3.06) and 18.59 (Std Dev: 2.53) in the sub-mSv group. Mean effective diameter of the SD cohort was 25.24 cm (Std Dev: 3.24) and 27.63 cm (Std Dev: 5.95) in the sub-mSv group. The median HI was 3.60 (Interquartile Range (IQR): 3.27–3.90) in the SD group compared to the sub-mSv group with a median of 3.40 (IQR: 2.65–4.00). Cohort characteristics are represented in Figure 2 and summarized in Table 1. There was a higher rate of modified Ravitch technique used in the SD group (20%) than the sub-mSv group (5%) as opposed to the more commonly performed Nuss procedure.

### Clinical Outcomes

**Figure 3:**
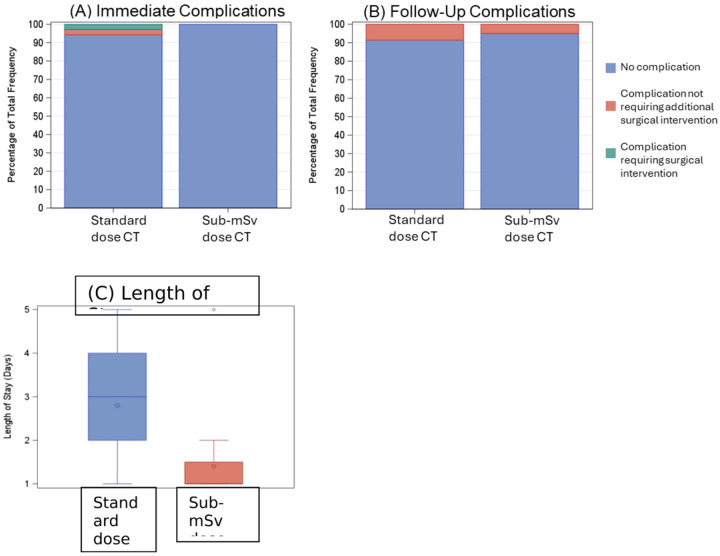
(A) Immediate post-surgical outcomes and (B) follow up outpatient outcomes. (C) Total length of hospital stays for SD and sub-mSv cohorts. Both clinical outcomes had similar distributions between the SD and sub-mSv cohorts, while length of stay was generally shorter for the sub-mSv cohort.

Two individuals in the SD group experienced post-operative chest wall hematomas during their inpatient stay. One individual (2.9%) required repeat operative intervention for evacuation. The second patient required a transfusion without additional operative re-intervention. No complications were observed in the sub-mSv chest CT cohort. Three individuals in the SD group (8.6%) had complications at follow-up meeting composite criteria which did not require further surgical intervention. There was only 1 individual in the sub-mSv group (5%) with an outcome in this category at follow-up. The average length of stay was 2.80 days (Std Dev: 1.11) for the SD cohort and 1.40 days (Std Dev: 0.94) for the sub-mSv cohort. Clinical outcomes are shown in Figure 3 and summarized in Table 3.

### CT Image Quality and Radiation Dose Metrics

No repeat CT scans were performed at the time of the initial scan due to image quality, and no patients were requested to return for repeat imaging due to non-diagnostic image quality. Haller indices and/or correction indices were successfully measured on all scans. Representative patients from both cohorts are shown in Figure 4. CTDI_vol_ was lower in the sub-mSv cohort with a median of 0.03 mGy (IQR: 0.03–0.04), compared to the SD group with a median of 4.63 mGy (IQR: 2.00–6.52). DLP was lower in the sub-mSv group with a median of 1.35 mGy*cm (IQR: 1.23–1.49); the SD group had a median of 186.60 mGy*cm (IQR: 75.80–246.00). SSDE was lower in the sub-mSv group with a median of 0.04 mGy (IQR: 0.04–0.06); the SD group had a median of 6.70 mGy (IQR: 3.20–9.54). Effective dose was higher in the SD group as expected, with a median of 2.43 mSv (IQR: 0.99–3.20), compared to the sub-mSv group with a median of 0.06 mSv (IQR: 0.05–0.06). SSDE and effective dose distributions are presented in Figure 5. Radiation dose metrics are summarized in Table 3.

### Statistical Analysis

All statistical test results are presented in Table 4. There was no evidence of differences between SD and sub-mSv CTs for age (unadjusted p=0.32, adjusted p=1), Haller index (unadjusted p=0.27, adjusted p=1), BMI (unadjusted p=0.44, adjusted p=1), effective diameter (unadjusted p=0.22, adjusted p=1) or either of the clinical outcomes (immediate: unadjusted and adjusted p=1; outpatient follow up: unadjusted and adjusted p=1).

There was a significant difference in length of stay between groups, with the sub-mSv group having shorter lengths of stay (Hodges-Lehmann estimate (95% C.I.): −2.00 (−2.00, −1.00); unadjusted and adjusted p < 0.001). There was also a significant difference in SSDE and effective dose between SD and sub-mSv CTs, with lower values in the sub-mSv group as expected (Hodges-Lehmann estimate (95% C.I.): SSDE −6.66 (−8.55, −4.08), effective dose −2.10 (−2.88, −1.28); unadjusted and adjusted p < 0.001 for both).

## Discussion

Introduction of a photon-counting detector CT (PCD-CT) at our institution enabled a natural comparison between two cohorts of patients undergoing evaluation and treatment for the same pathology. All patients with congenital pectus deformity in this study were evaluated and treated in the same clinical setting. Outcomes at the one-month postoperative visit served as the main study endpoint in keeping with quality metrics tracked by the American College of Surgeons National Surgical Quality Improvement Program (ACS-NSQIP). Demographic and clinical characteristics including age, BMI, effective diameter, and HI, did not differ significantly between groups. Rates of postoperative complications also did not differ significantly across cohorts and were consistent with published literature [27, 28].

Radiation dose differences between cohorts were substantial with the dose for the sub-mSV cohort comparable to conventional chest radiography [29]. Importantly, none of the sub-mSv scans were deemed non-diagnostic by interpreting radiologists despite the low radiation doses used. Osseous structures of the chest were clearly visualized against low-attenuation soft tissues. Prior studies have similarly demonstrated the adequacy of low-dose CT for evaluating the bones of the thorax [24, 30, 31]. Importantly, these findings demonstrate that significant radiation dose reductions can be achieved without compromising diagnostic quality for preoperative evaluation of congenital osseous abnormalities. The primary hypothesis that sub-mSv non-contrast chest CT does not adversely affect clinical outcomes in the assessment and surgical management of congenital chest wall deformities is not rejected by our data.

Our results revealed a statistically significant reduction in immediate postoperative length of stay in the sub-mSv cohort as compared to the SD cohort. While numerous factors influence length of stay outcomes, we do not attribute this difference to the sub-mSv dose CT acquisition. There was an evolution in analgesic strategy during the study period where patients initially received no anesthetic blocks, then regional catheters, and finally intercostal nerve cryoablation [32]. The cryoablation has significantly shortened the length of stay for these patients and is beyond the scope of this study.

Limitations of our study include the retrospective nature of data gathering as well as the treatment and diagnosis of the disease process. Retrospective nature of our data is limited by the lack of a sub-mSv protocol on our EID-CT scanners. Prior investigations have made similar comparisons between control EID-CT and experimental PCD-CT groups; however, further investigation remains needed into dose reduction and outcomes across multiple scanner generations [33, 34]. Electronic noise contributes more significantly to the degradation of image quality as CT radiation doses are reduced approaching that of chest radiography. The inherent ability of PCD-CTs to remove electronic noise permitted development of new protocols presented above while sustaining a degree of acceptable image quality. The protocols developed in this study serve to inform future exploration of clinical applications using PCD-CT in pediatric chest imaging. Sample size was limited in this study and had low complication rates in both groups. The single institution design captured the volume at our tertiary referral academic hospital which correlates but is also subject to overall disease incidence amongst the population. While an equivalence or non-inferiority framework may be better suited to investigate comparisons in complication rates, we were limited with the sample size, and it is difficult to determine the margin for equivalence or non-inferiority. The single pre-operative scan and single surgery nature of congenital chest wall deformity treatment makes repeated imaging using different control and experimental protocols impractical.

Patient age, size (BMI and effective diameter), and chest wall size (HI) were compared in this study without substantial differences. Future studies should consider matching (or propensity score matching) and/or utilizing an equivalence or non-inferiority framework if sample sizes allow; our results can be used to inform such studies.

Recent reports linking pediatric radiation exposure to increased malignancy risk underscore the importance of continued dose-reduction efforts [35]. This study contributes to the growing body of literature on image quality and radiation dose reduction using PCD-CT. While prior research has focused primarily on technical performance and subjective image quality, this study also considered clinical outcomes. Our data reinforces the imperative that aggressive dose-reduction strategies can be achieved without compromising the quality of patient care for certain applications and highlights the need for continued investigation into optimizing radiation doses for pediatric patients.

## Conclusions

Radiation doses from non-contrast chest CT performed for preoperative planning of congenital pectus deformity can be substantially reduced without compromising surgical outcomes. In this study, the pre-operative examinations performed on the photon-counting detector CT (PCD-CT) utilized dose levels comparable to modern chest radiography while preserving the necessary diagnostic image quality. Further investigation is warranted to expand the clinical applications of this technology, particularly in radiosensitive pediatric populations.

## Figures and Tables

**Table 1.** Participant demographics between standard dose CT and sub-mSv dose CT cohorts. Summary of patient pre-operative characteristics including Haller Index, BMI, and effective diameter.

	Standard Dose CTs (N=35)	Sub-mSv CTs (N=20)	Total (N=55)
**Sex**			
Female	2 (5.7%)	4 (20.0%)	6 (10.9%)
Male	33 (94.3%)	16 (80.0%)	49 (89.1%)
**Age at Time of CT**			
Mean (Std Dev)	15.96 (1.98)	15.34 (1.75)	15.74 (1.91)
Median	16.27	15.14	15.45
Q1, Q3	14.21, 17.62	14.11, 16.67	14.18, 17.02
Range	(12.94–19.79)	(12.55–19.00)	(12.55–19.79)
**Haller Index**			
Mean (Std Dev)	3.86 (1.23)	3.57 (1.38)	3.76 (1.28)
Median	3.60	3.40	3.55
Q1, Q3	3.27, 3.90	2.65, 4.00	3.00, 3.90
Range	(2.60–8.90)	(1.50–8.10)	(1.50–8.90)
**BMI**			
Mean (Std Dev)	19.22 (3.06)	18.59 (2.53)	18.99 (2.87)
Median	18.90	18.53	18.79
Q1, Q3	16.74, 21.20	16.47, 20.59	16.70, 20.88
Range	(14.54–27.50)	(13.81–22.81)	(13.81–27.50)
**Effective Diameter**			
Mean (Std Dev)	25.24 (3.24)	27.63 (5.95)	26.11 (4.51)
Median	25.23	26.14	25.68
Q1, Q3	22.01, 26.77	23.53, 29.89	23.00, 27.38
Range	(19.84–34.61)	(19.08–40.88)	(19.08–40.88)

**Table 2.** Sub-mSv non-contrast chest CT protocol for PCD-CT.

Scan Parameters	
Scan Mode	Dual source helical
Detector configuration	144 × 0.4 mm
Tube potential (kV)	Fixed, 100 + Tin filter
Pitch	3.2
Rotation time (ms)	250
Effective mAs	5 (fixed)


Reconstruction Parameters				
Series	Thick	Thin	Lung	MIP
Slice thickness (mm)	3.0	0.4	0.4	7
Iterative Reconstruction Strength	3	3	1	3
Reconstruction Energy (keV)	T3D	73	73	73
Kernel	Br44	Br44	Qr44	Br44

**Table 3.** Results summary of patient population characteristics, surgical outcomes, length of stay, and CT radiation dose profile.

	Standard Dose CTs (N=35)	Sub-mSv CTs (N=20)	Total (N=55)
**Immediate Clinical Outcome**			
No Complication	33 (94.3%)	20 (100.0%)	53 (96.4%)
Mild Complication	1 (2.9%)	0 (0.0%)	1 (1.8%)
Moderate/Severe Complication	1 (2.9%)	0 (0%)	1 (1.8%)
**Follow Up Clinical Outcome**			
No Complication	32 (91.4%)	19 (95.0%)	51 (92.7%)
Mild Complication	3 (8.6%)	1 (5.0%)	4 (7.3%)
Moderate/Severe Complication	0 (0%)	0 (0%)	0 (0%)
**Length of Stay (Days)**			
Mean (Std Dev)	2.80 (1.11)	1.40 (0.94)	2.29 (1.24)
Median	3.00	1.00	2.00
Q1, Q3	2.00, 4.00	1.00, 1.50	1.00, 3.00
Range	(1.00–5.00)	(1.00–5.00)	(1.00–5.00)
**CT Dose Index (CTDI**_**voI**_ **)**			
Mean (Std Dev)	4.56 (2.33)	0.10 (0.16)	2.94 (2.85)
Median	4.63	0.03	1.98
Q1, Q3	2.00, 6.52	0.03, 0.04	0.03, 5.80
Range	(1.54–9.93)	(0.03–0.55)	(0.03–9.93)
**Dose Length Product (DLP)**			
Mean (Std Dev)	179.64 (93.19)	3.78 (5.76)	115.69 (113.00)
Median	186.60	1.35	74.50
Q1, Q3	75.80, 246.00	1.23, 1.49	1.42, 225.10
Range	(57.00–415.80)	(1.06–20.80)	(1.06–415.80)
**Size Specific Dose Estimate (SSDE)**			
Mean (Std Dev)	6.61 (3.17)	0.12 (0.15)	4.25 (4.03)
Median	6.70	0.04	3.09
Q1, Q3	3.20, 9.54	0.04, 0.06	0.05, 8.51
Range	(1.74–12.00)	(0.03–0.47)	(0.03–12.00)
**Effective Dose (mSv)**			
Mean (Std Dev)	2.34 (1.21)	0.16 (0.25)	1.55 (1.43)
Median	2.43	0.06	0.97
Q1, Q3	0.99, 3.20	0.05, 0.06	0.06, 2.93
Range	(0.74–5.41)	(0.05–0.87)	(0.05–5.41)

**Table 4.** Testing results for comparison between SD and sub-mSv cohorts.

Variable	Location Shift Estimate (95% C.I.) *^$^	Unadjusted P-Value^†^	Adjusted P-Value^‡^
Age	−0.52 (−1.71, 0.59)	0.32	1
Haller Index	−0.30 (−0.80, 0.20)	0.27	1
BMI	−0.63 (−2.25, 0.99)	0.44	1
Effective Diameter	1.22 (−0.74, 3.91)	0.22	1
Immediate Clinical Outcome	-	1	1
Follow Up Clinical Outcome	-	1	1
Length of Stay	−2.00 (−2.00, −1.00)	<0.001	<0.001
Size Specific Dose Estimate	−6.66 (−8.55, −4.08)	<0.001	<0.001
Effective Dose	−2.10 (−2.88, −1.28)	<0.001	<0.001

* Hodges-Lehmann estimates (age, Haller index, effective diameter, length of stay, size specific dose estimate, effective dose), difference in means (BMI);

$ difference in sub-mSv minus SD.

† P-values from Fisher’s exact test (clinical outcomes), Wilcoxon rank sum tests (age, Haller index, effective diameter, length of stay, size specific dose estimate, effective dose), two-sample t-test with equal variance (BMI).

‡ Adjusted p-values using Holm method.

## Data Availability

All data supporting the findings of this study are available within the paper.
